# Supplementary material for: Real-time visualization of clustering and intracellular transport of gold nanoparticles by correlative imaging
Source: Nat Commun. 2017 May 31;8:15646. doi: 10.1038/ncomms15646 (PMC5460036; doi:10.1038/ncomms15646)
Supplement: Supplementary Information — Supplementary Figures, Supplementary Discussion, Supplementary Methods and Supplementary References. [file ncomms15646-s1.pdf]

Supplementary Figures

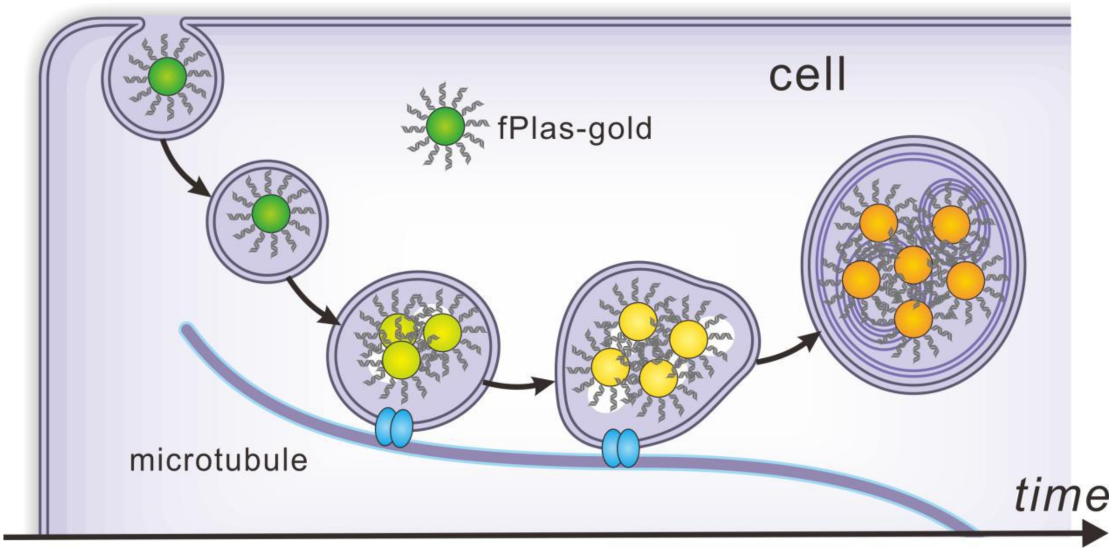

Supplementary Figure 1. Schematic illustration of clustering of fPlas-gold during the intracellular traffic along microtubules. fPlas-gold nanoparticles enter cells mainly in the monomeric form and those trapped in early endosomes are gradually clustered via vesicle fusion during the maturation process. fPlas-gold nanoparticles in lysosomes exist predominantly as large clusters.

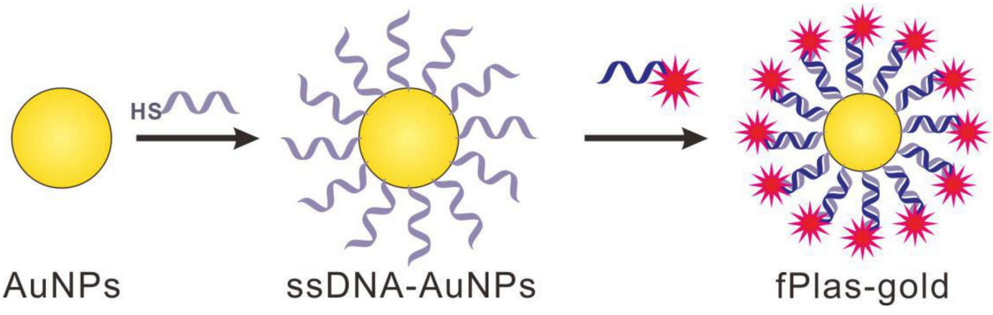

Supplementary Figure 2. Preparation of fPlas-gold for fluorescence microscopy (FM) and dark-field microscopy (DFM).

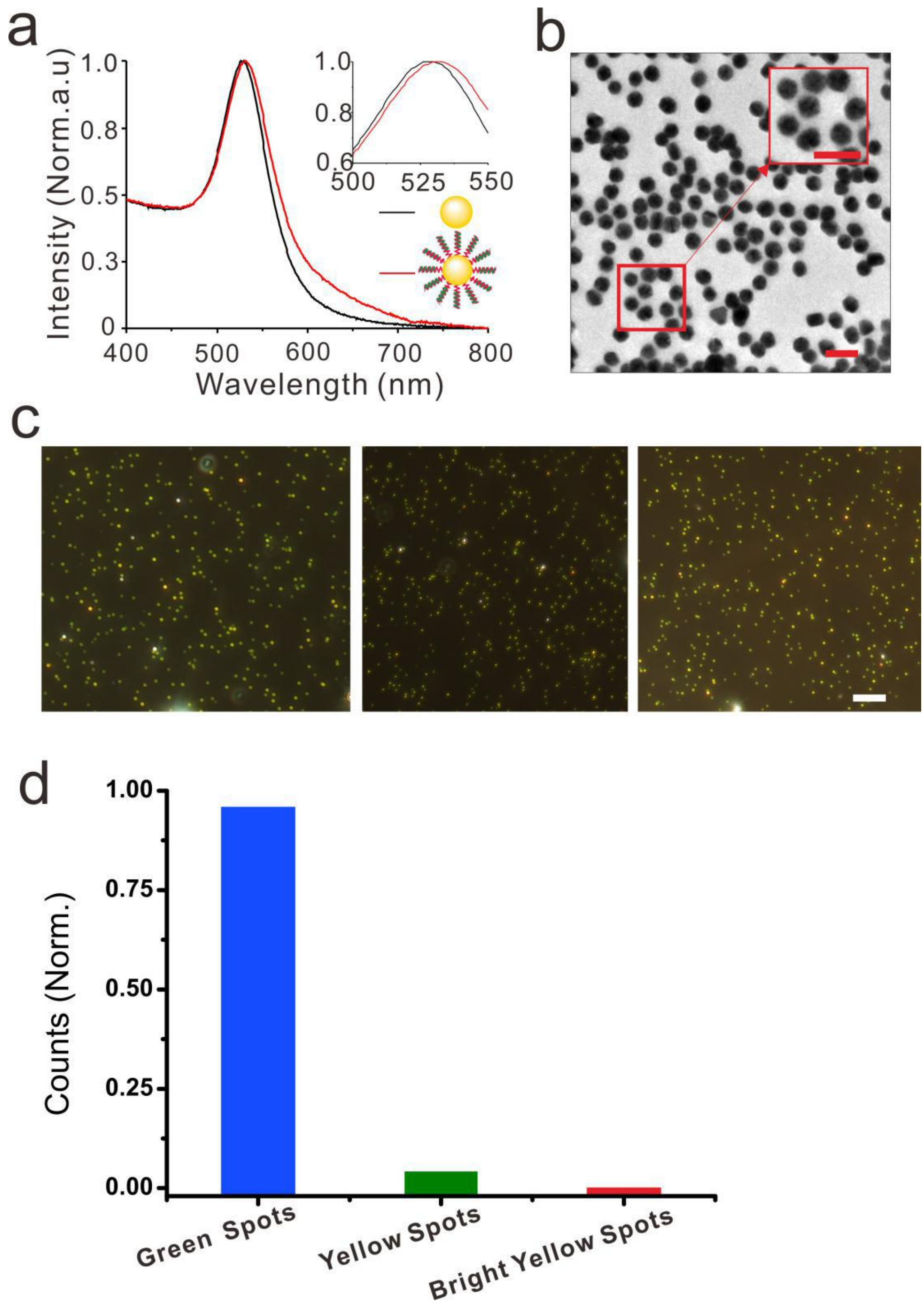

Supplementary Figure 3. Characterization of fPlas-gold. (a) Nanoparticle absorbance spectra before (black) and after (red) DNA modification. (b) A representative TEM image (Scale bar represents 100 nm) and (c) three representative DFM images (Scale bar represents 20 μm) of fPlas-gold. (d) Over 1,000 dots in DFM

images were analyzed, revealing that more than 95% of fPlas-gold in water on glass existed as green dots in DFM images.

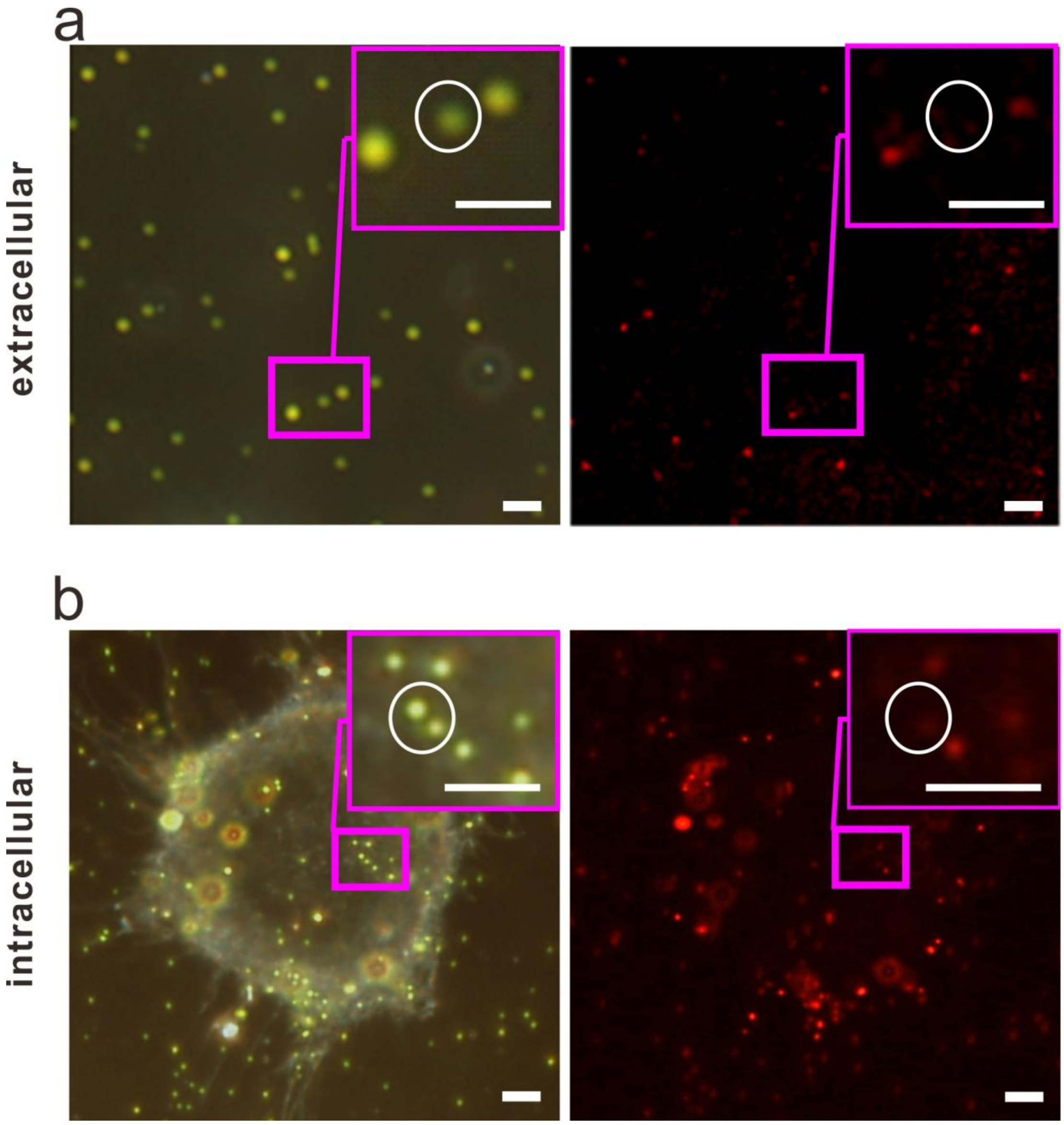

Supplementary Figure 4. Representative DFM and FM images of extracellular and intracellular fPlas-gold in the same field of view. Inserted pictures were zoomed in pictures of the area in pink rectangles. Both DFM images and FM images of fPlas-gold were taken on the correlative microscope. Scale bar represents 5 μm.

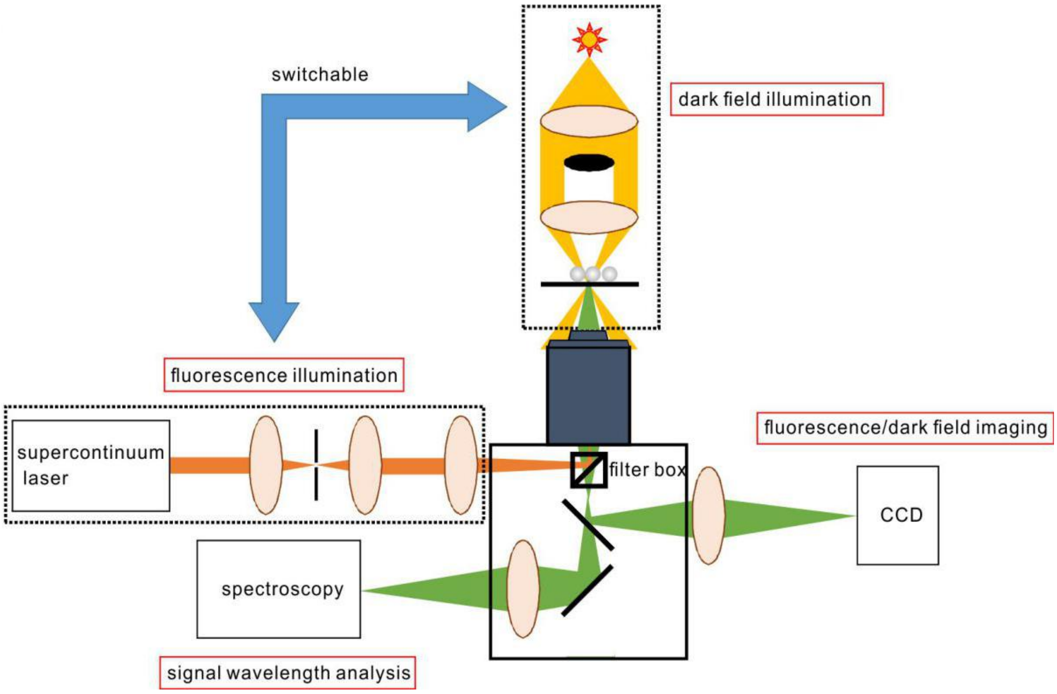

Supplementary Figure 5. Schematic illustration of the optical pathway diagram of the correlative microscope employed in the present study.

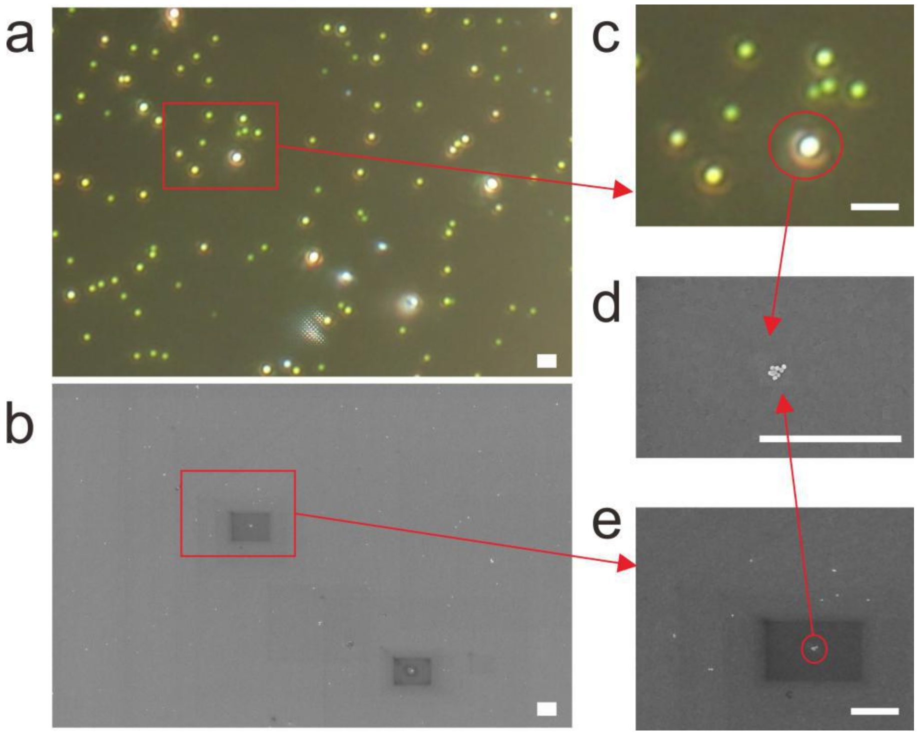

Supplementary Figure 6. Determination of fPlas-gold aggregation states. (a) A

wide-field DFM image containing approximately 100 spots of different colors. (b) The ex-situ SEM image of the fPlas-gold particles recorded in a. The red rectangular area in a and b were zoomed in as shown in (c) and (e), respectively, in which the bright yellow spot in the red circle contained 8 fPlas-gold single particles as indicated in (d). Scale bar represents 2 μm.

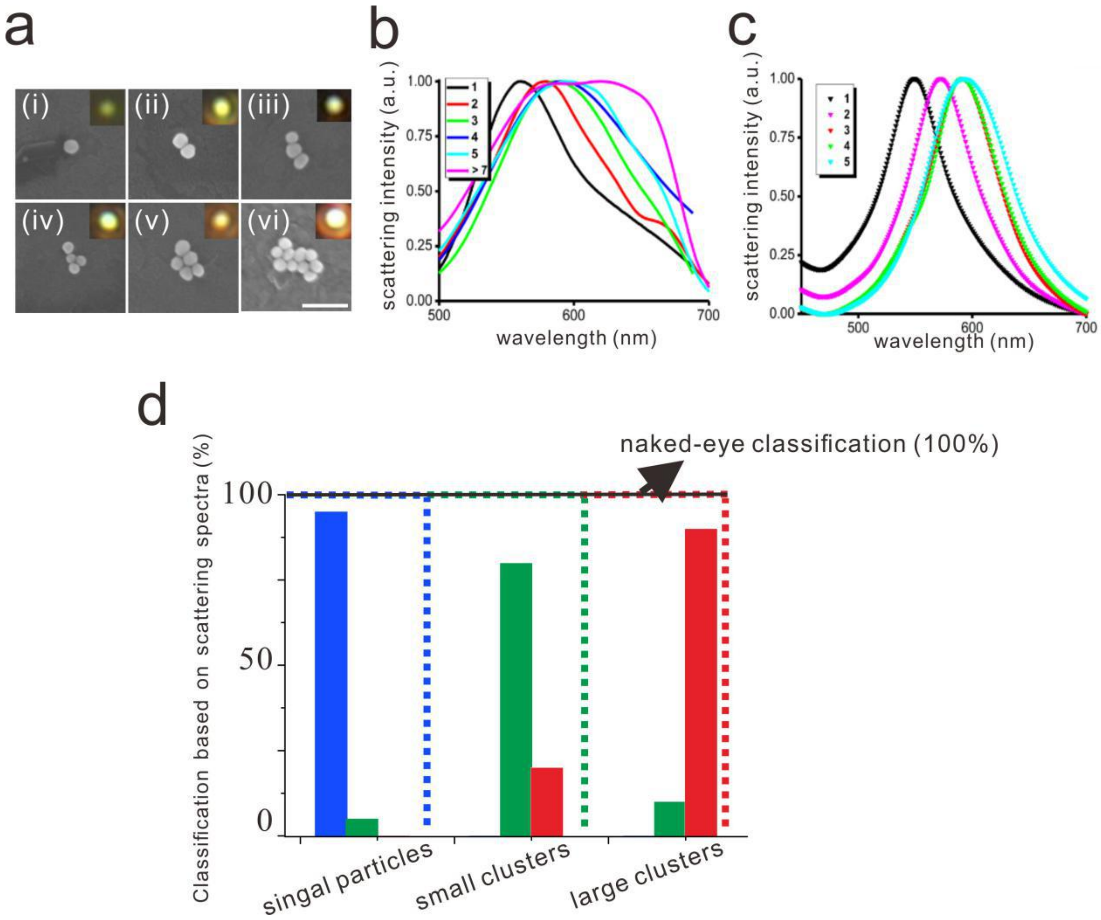

Supplementary Figure 7. Optical property of fPlas-gold. (a) Representative SEM images of clusters of different sizes and the corresponding DFM images. Scale bar represents 200 nm. (b) Scattering spectra detected by experiment and (c) simulated by FDTD for fPlas-gold shown in a. The scattering spectra gradually shifted to the red with the increase of cluster size. (d) We compared naked-eye classification of randomly-selected 20 fPlas-gold clusters with the results based on scattering spectra, and found a good agreement.

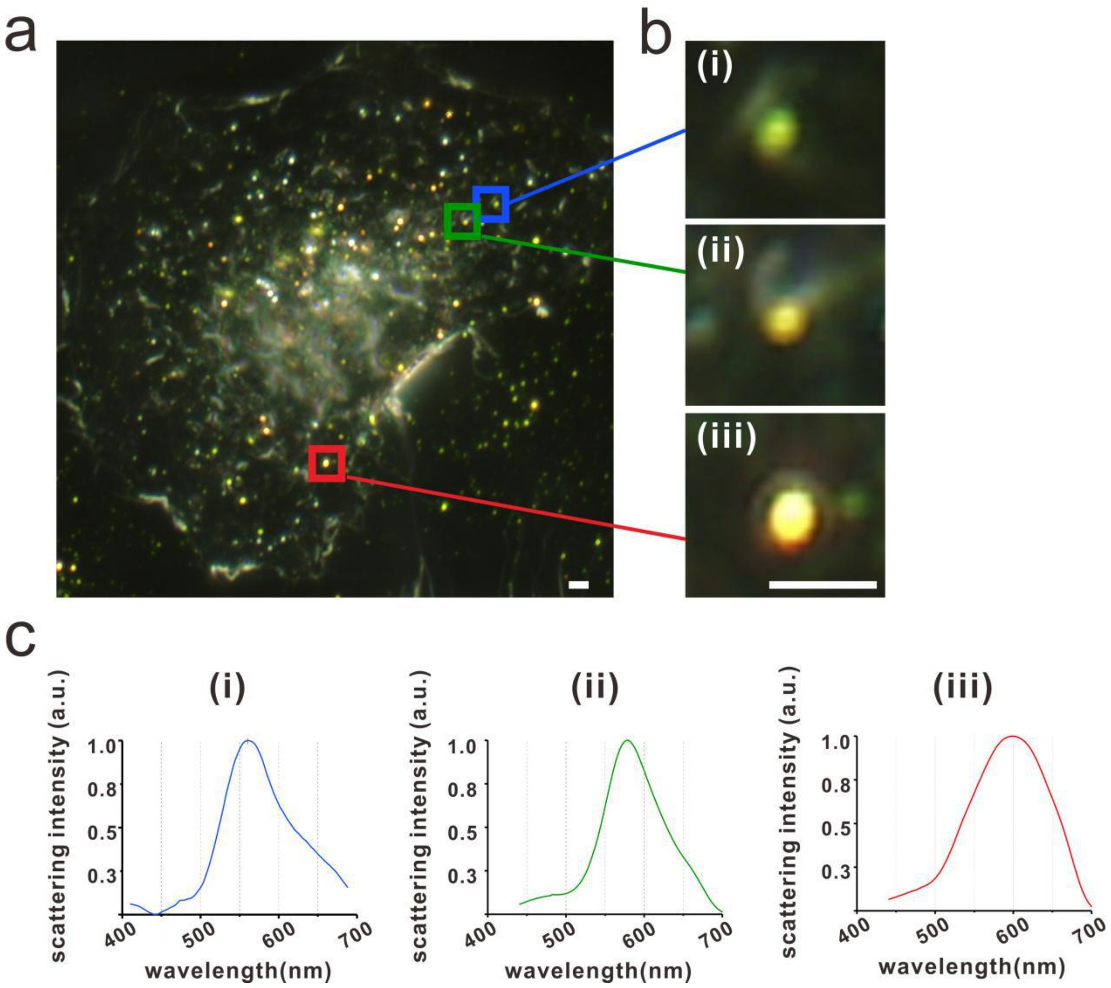

Supplementary Figure 8. Representative examples of (a and b) DFM images and (c) scattering spectra of intracellular fPlas-gold. Green (i), yellow (ii) and bright yellow (iii) dots in the DFM image represented single particles (n=1), small clusters (n=2-5) and large clusters (n>5), respectively. The scattering spectra of the three intracellular fPlas-gold nanoparticles were distinguishable, which shifted from 560 nm to 600 nm with the increase of cluster size. Scale bar represents 2 μm.

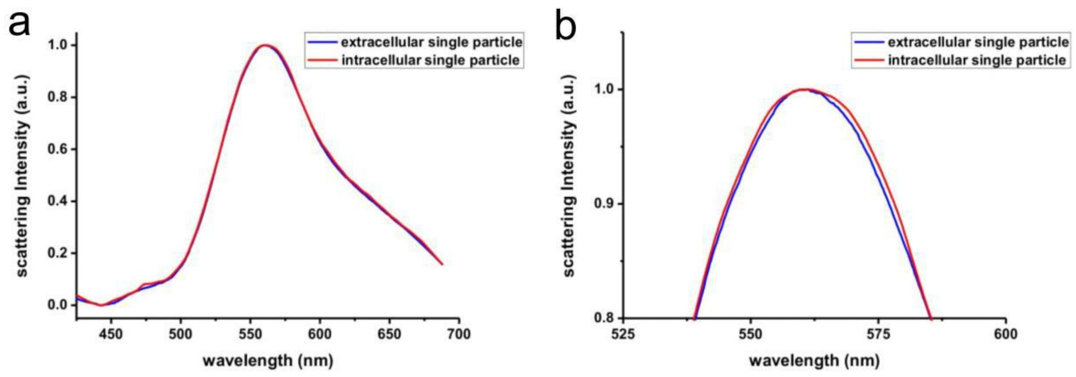

Supplementary Figure 9. Comparison of light scattering spectra of extracellular and intracellular single particle fPlas-gold. The scattering spectra of the green dot in Supplementary Figure 7a-i (extracellular, blue line) and Supplementary Figure 8b-i (intracellular, red line) matched well. Figure (b) was the zoomed-in spectra of (a).

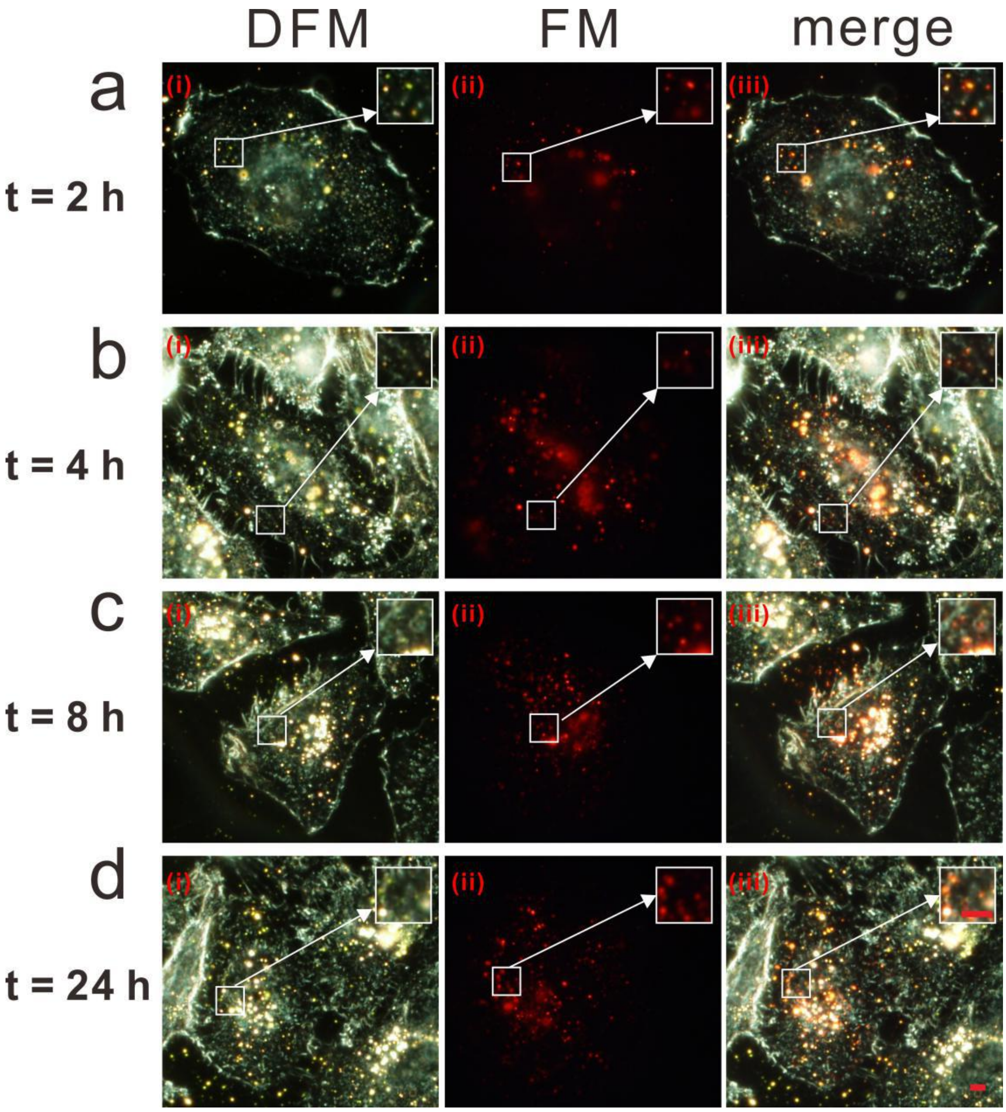

Supplementary Figure 10. Time-dependent cellular uptake of fPlas-gold. Representative examples of DFM images of HeLa cells incubated with 0.1 nM fPlas-gold for different time. Both DFM images and FM images were taken by using the correlative microscope. Scale bar represents 2 μm.

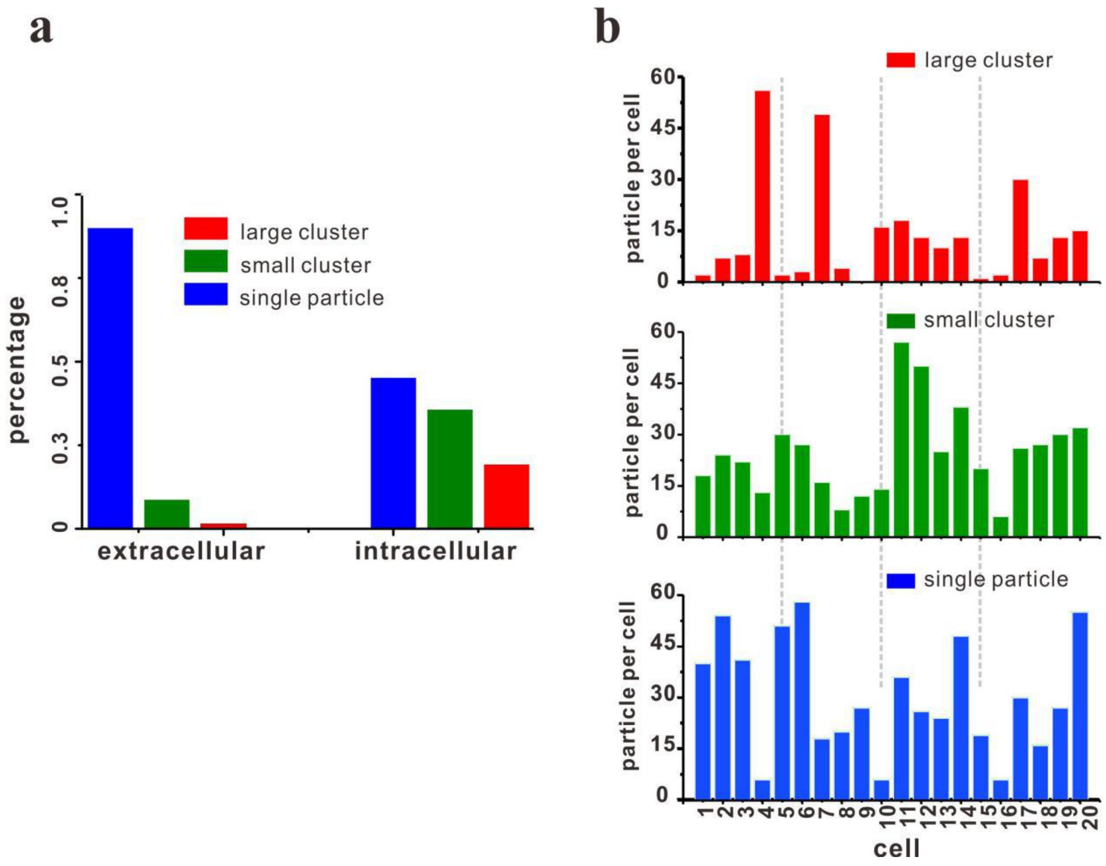

Supplementary Figure 11. Different aggregation states of extracellular and intracellular fPlas-gold. (a) Percentages of single particles, small clusters and large clusters in 1000 randomly selected spots in and out of cells, respectively. (b) Single-cell analysis of distribution of single particles, small clusters and large clusters, indicating the aggregation of intracellular fPlas-gold is cell dependent. 20 cells were analyzed.

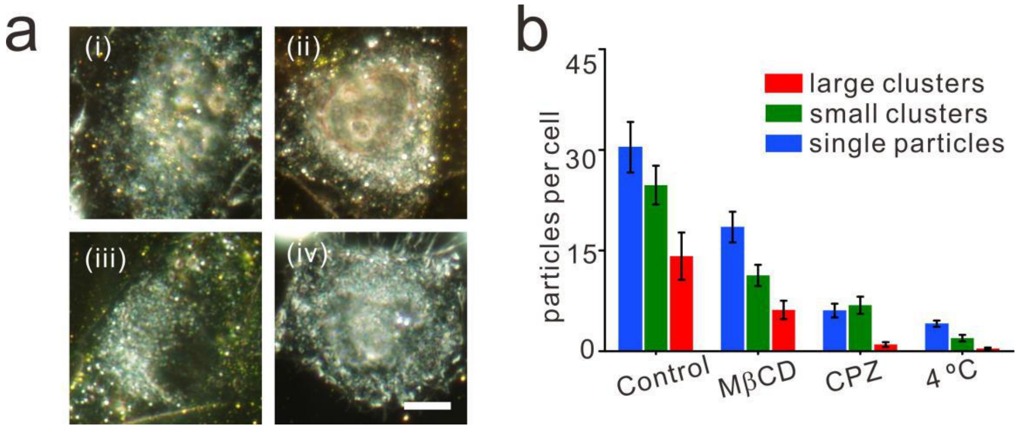

Supplementary Figure 12. Cell internalization of fPlas-gold employing different temperatures and pharmacological inhibitors. (a) Representative DFM images of HeLa cells without treatment (i) and incubated with 2.5 mM MβCD (ii), 10 μg mL^{-1} chlorpromazine (iii) and cultured at 4 °C (iv). (b) Averaged numbers of different fPlas-gold clusters in 20 cells. Data were presented as the mean ± SEM. Scale bar represents 10 μm.

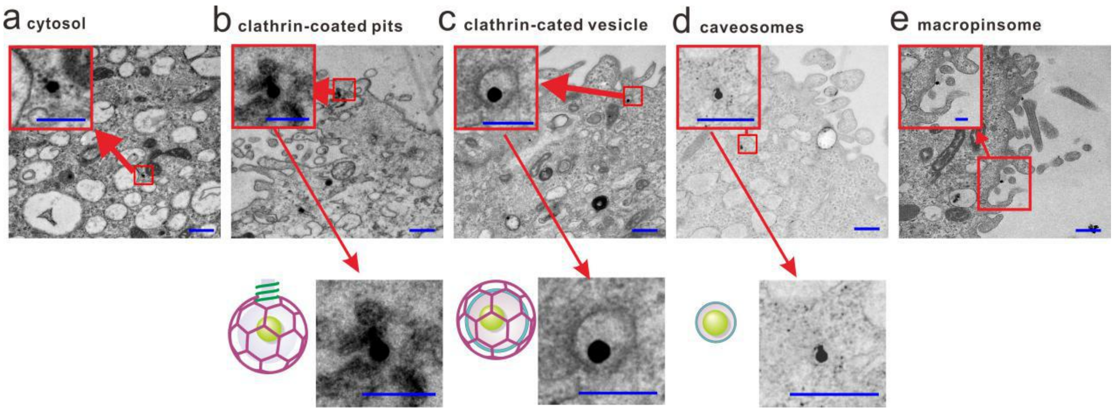

Supplementary Figure 13. Representative examples of TEM images indicating fPlas-gold were localized in various organelles. (a) One single particle was located in cytosol; (b) two single particles were wrapped by a coated pit connected with cell surface by a neck, which was supposed to be a clathrin-coated vesicle; (c) a single particle was located in a coated vesicle with outer diameter of ~100 nm, which was also supposed to be a clathrin-coated vesicle; (d) a single particle was wrapped by a sphere-shape structure with outer diameter of ~70 nm without an electron-dense coat, which was supposed to be a caveolae; (e) a single particle located in a ruffle with diameter >1 μm, which was a macropinsome. Scale bar represents 200 nm.

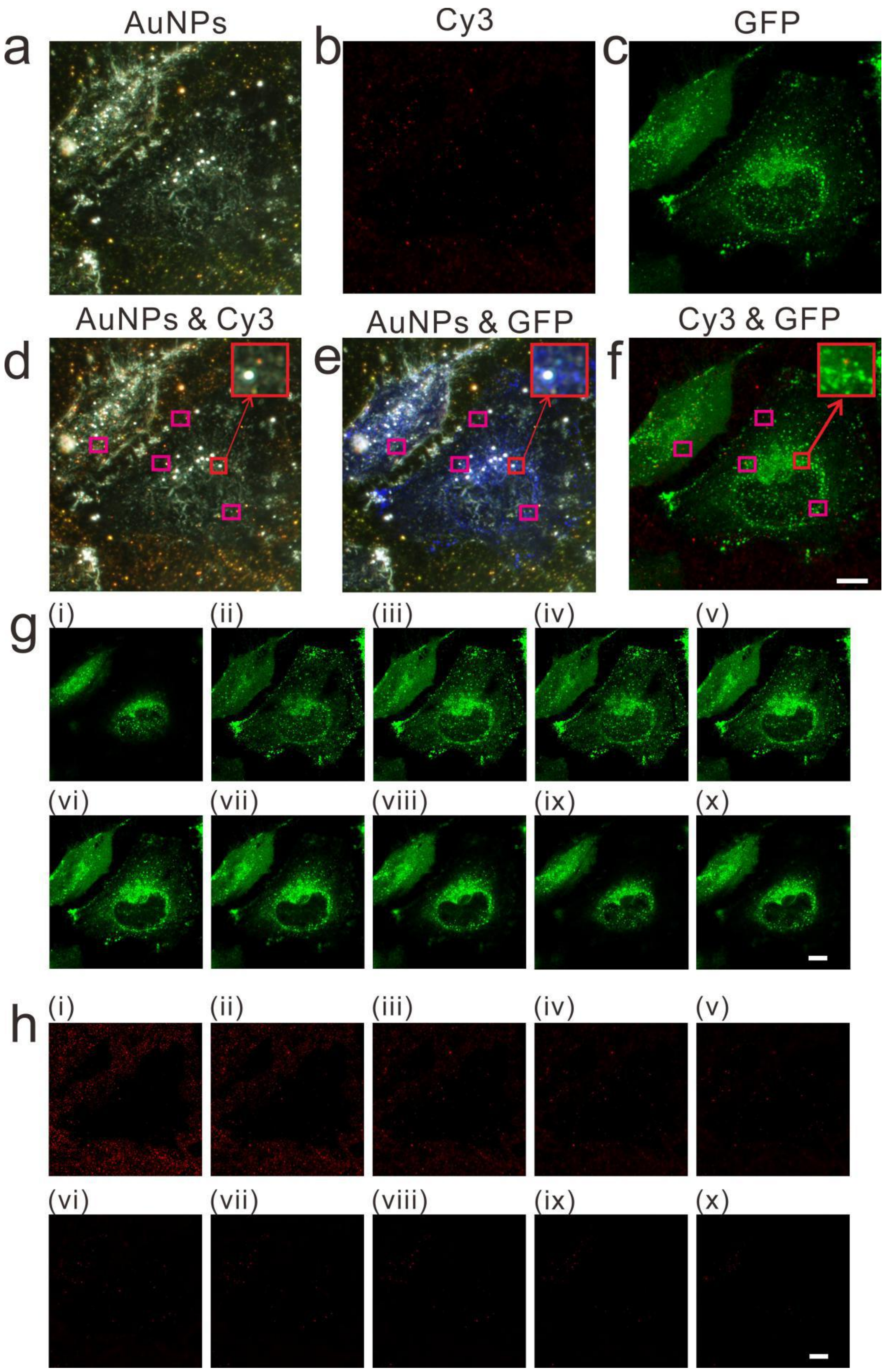

Supplementary Figure 14. Colocalization of fPlas-gold with GFP-fused early endosomes as revealed with DFM and FM imaging. (a) DFM image of fPlas-gold;

FM image of (b) fPlas-gold and (c) early endosomes; (d) overlay of a and b; (e) overlay of a and c; (f) overlay of b and c. To avoid influence of green GFP fluorescence on DFM signal of green spots, the green fluorescence was converted to blue color in e. Confocal 3D sectioning images of GFP-fused early endosomes (g) and fPlas-gold (h). Note: DFM images of fPlas-gold (a) were taken on the correlative microscope. FM images of fPlas-gold (b) and GFP-fused early endosomes (c) were taken on the confocal microscope layer by layer (see images g and h) and then reconstructed. Scale bar represents 10 μm.

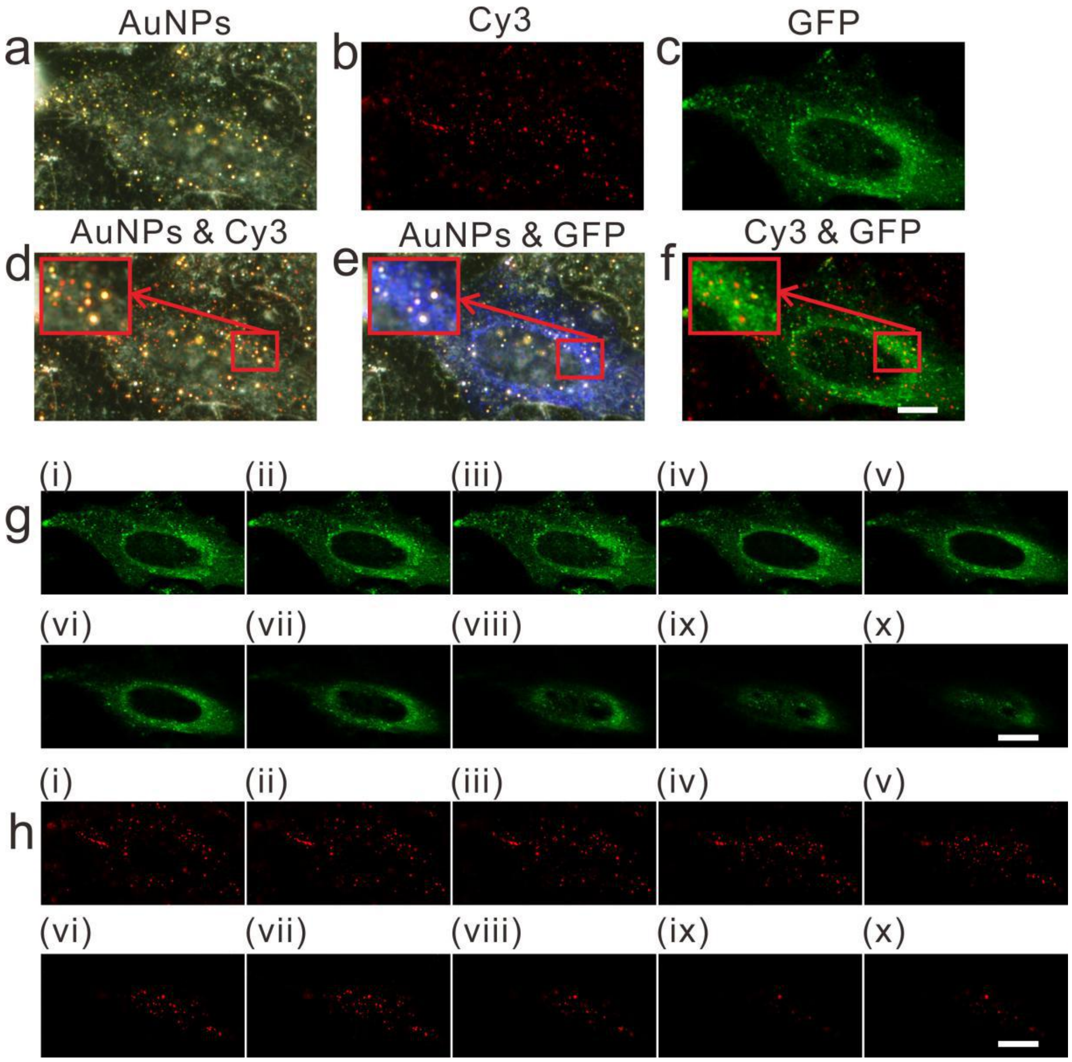

Supplementary Figure 15. Colocalization of fPlas-gold with GFP-fused late endosomes as revealed with DFM and FM imaging. (a) DFM image of fPlas-gold; FM image of (b) fPlas-gold and (c) early endosomes; (d) overlay of a and b; (e) overlay of a and c; (f) overlay of b and c. To avoid influence of green GFP fluorescence on DFM signal of green spots, the green fluorescence was converted to blue color in e. Confocal 3D sectioning images of GFP-fused late endosomes (g) and fPlas-gold (h). Note: DFM images of fPlas-gold (a) were taken on the correlative microscope. FM images of fPlas-gold (b) and GFP-fused late endosomes (c) were taken on the confocal microscope layer by layer (see images g and h) and then reconstructed. Scale bar represents 10 µm.

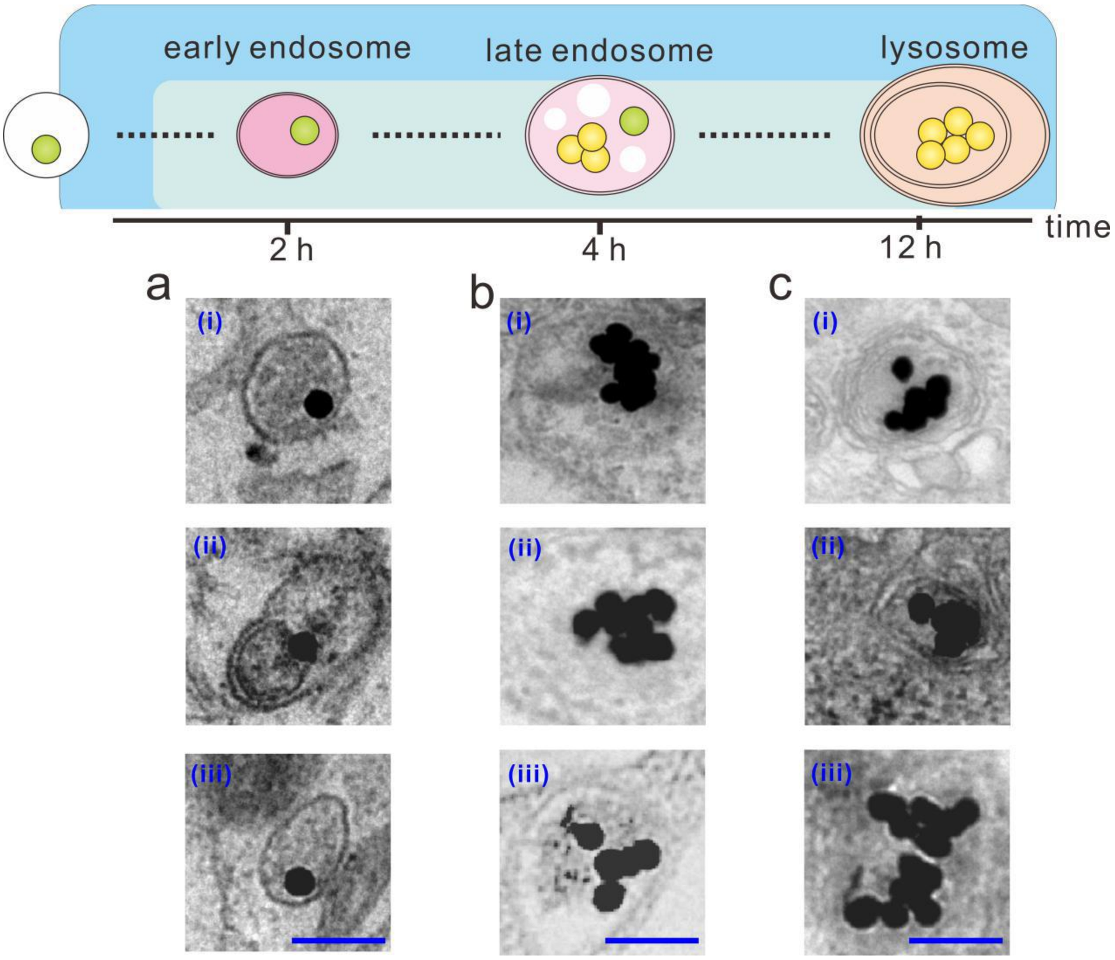

Supplementary Figure 16. Representative TEM images indicating fPlas-gold were localized in early endosomes (a), late endosomes (b) and lysosomes (c). In early endosomes, fPlas-gold were mainly single particles, while in late endosomes and lysosomes they were mainly small clusters and large clusters, respectively. This observation strongly suggested clustering of fPlas-gold occurred during the vesicle transport and maturation. Scale bar represents 200 nm.

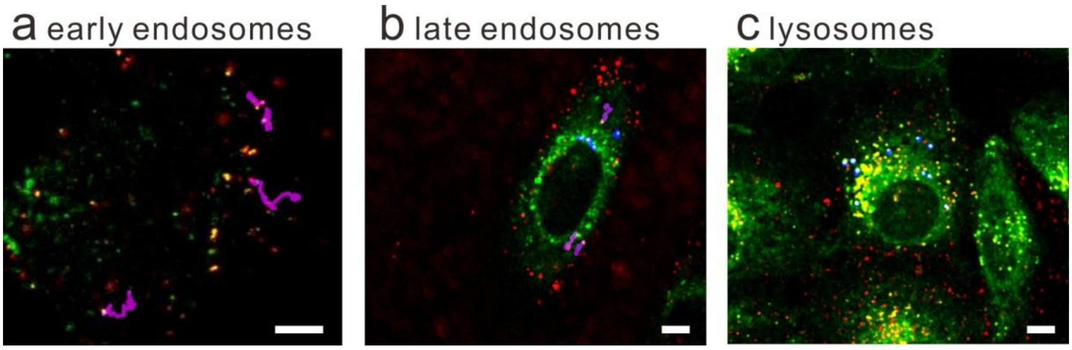

Supplementary Figure 17. Representative trajectories of intracellular fPlas-gold of high mobility (magenta) and low mobility (blue) in FM images. See Supplementary Movie 2-4: Δt = 3 s, total time = 90 s for 2 (early endosomes) and 3 (late endosomes), 450 s for 4 (lysosomes). Scale bar represents 5 μm.

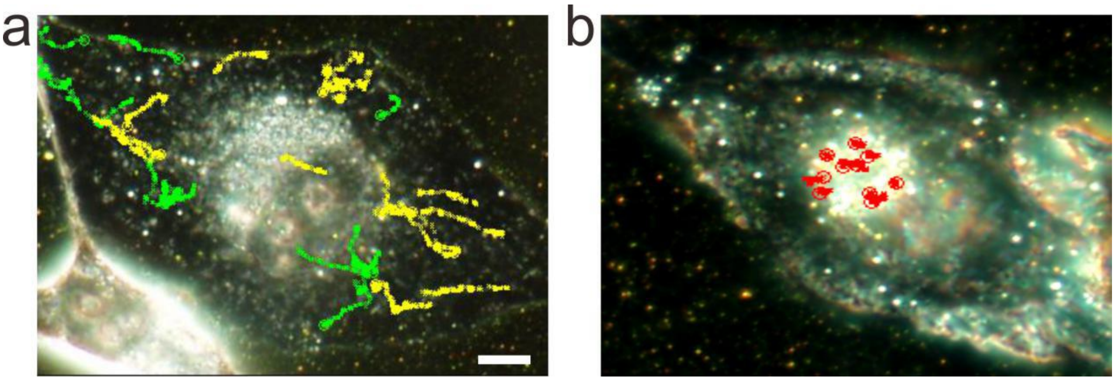

Supplementary Figure 18. Representative trajectories of intracellular single particles (green), small clusters (yellow) and large clusters (red) in DFM images. See Supplementary Movie 9 and 10: Δt = 1s, total time = 300 s. Scale bar represents 5 μm.

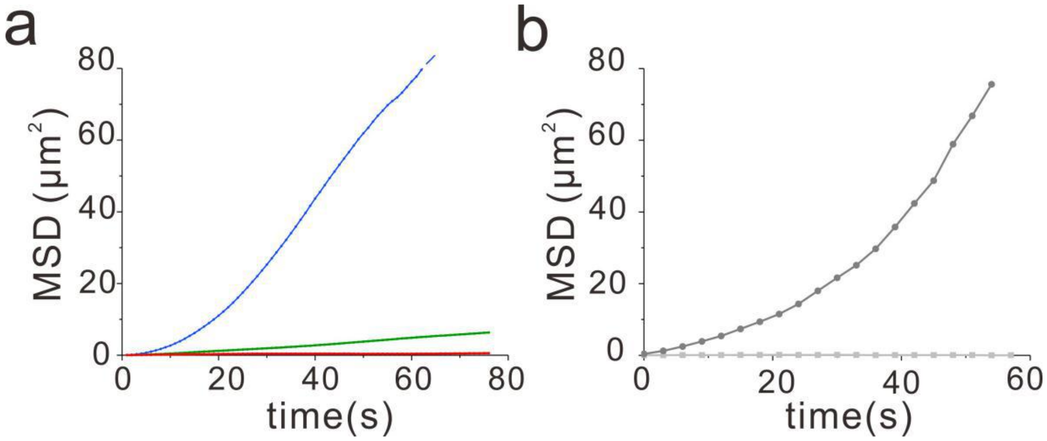

Supplementary Figure 19. 2D mean square displacement (MSD) analysis of fPlas-gold movement shown in Figure 5. Particles observed using DFM (a) were classified as single particles (blue), small clusters (green) and large clusters (red) while the ones observed using FM (b) were classified as high-mobility (70% black) and low-mobility (20% black) ones. Data were collected from 20 spots in three independent experiments for each group.

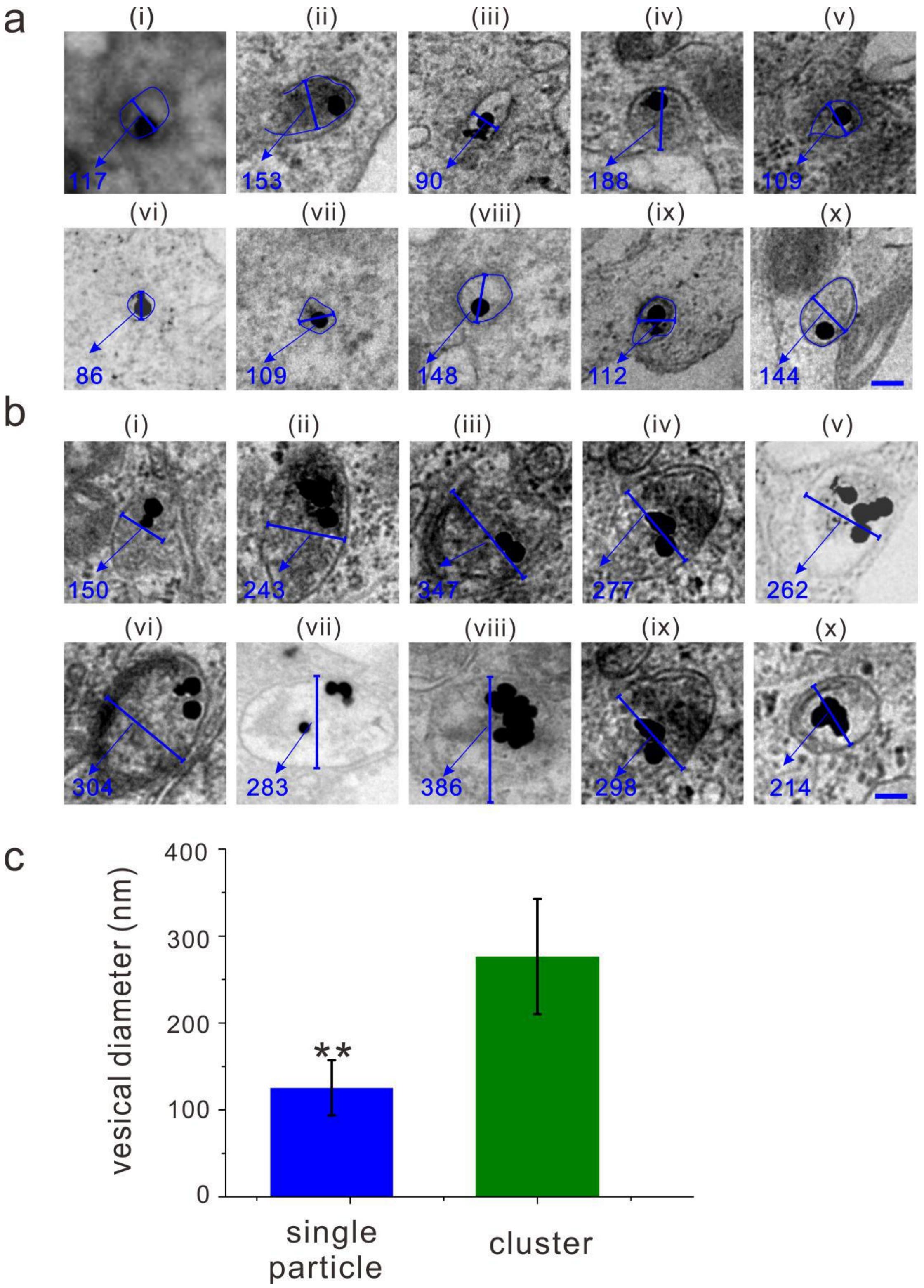

Supplementary Figure 20. Dependence of vesicle sizes on the number of encapsulated AuNPs. TEM images of randomly selected (a) ten vesicles containing single particles and (b) ten vesicles containing clusters. (c) The averaged vesicle diameters of single particles and clusters. Data were presented as the mean±SEM.

**P<0.01, according to two-tailed two-sample *t* test. Scale bar represents 100 nm.

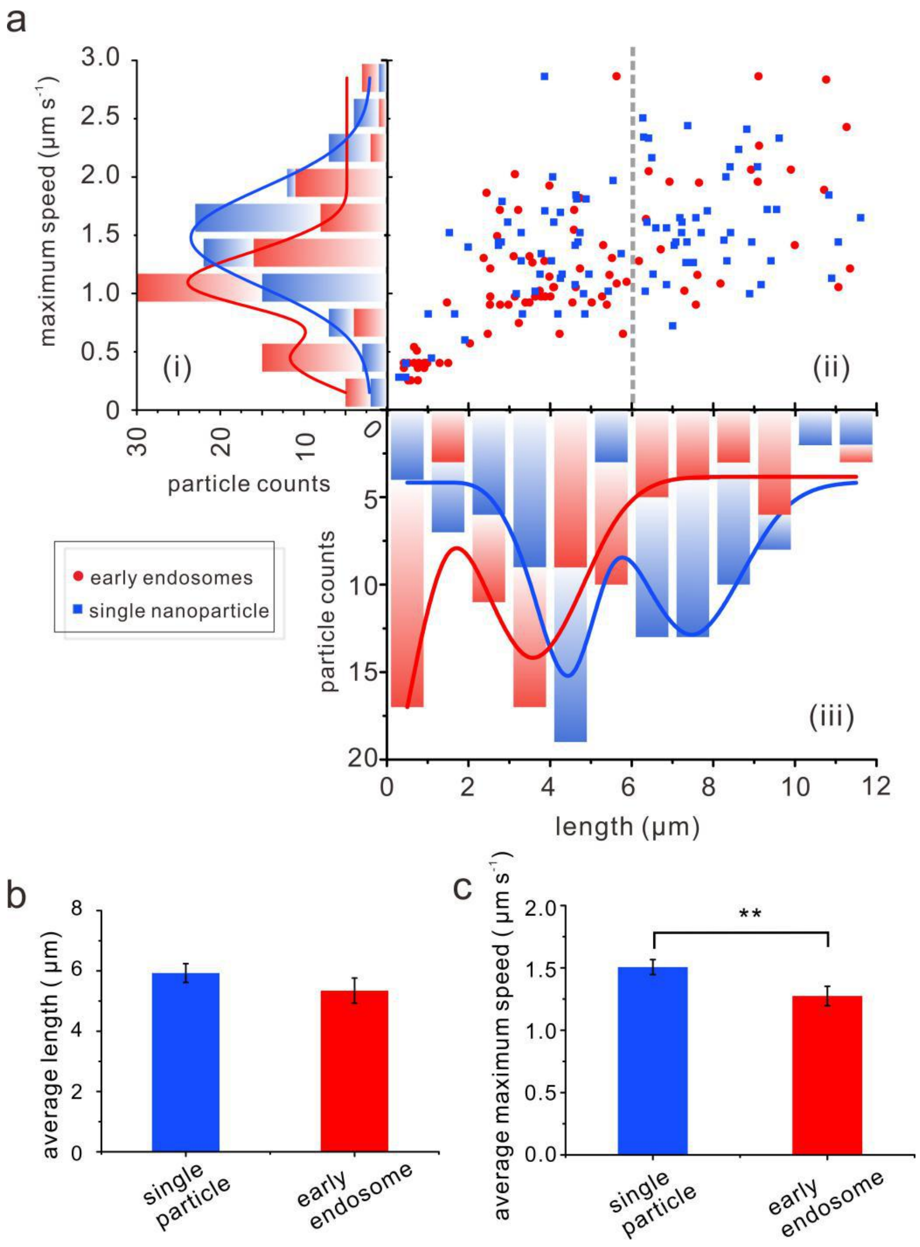

Supplementary Figure 21. Movement of single fPlas-gold nanoparticles and early endosomes in cells. (a-ii) Scatter plots showing passage length and maximum of frame to frame instantaneous speed for each single particle (blue) and early endosome (red); (a-i) histogram showing the distribution of speed; (a-iii) histogram showing the distribution of passage length. (b) Average passage length and (c) average maximum speed of single particles and early endosomes (data obtain from single-particle analysis shown in (a), and were presented as the mean ± SEM. **P<0.01, according to two-tailed two-sample *t* test). Data were collected from 100 randomly selected spots

in three independent experiments for each group.

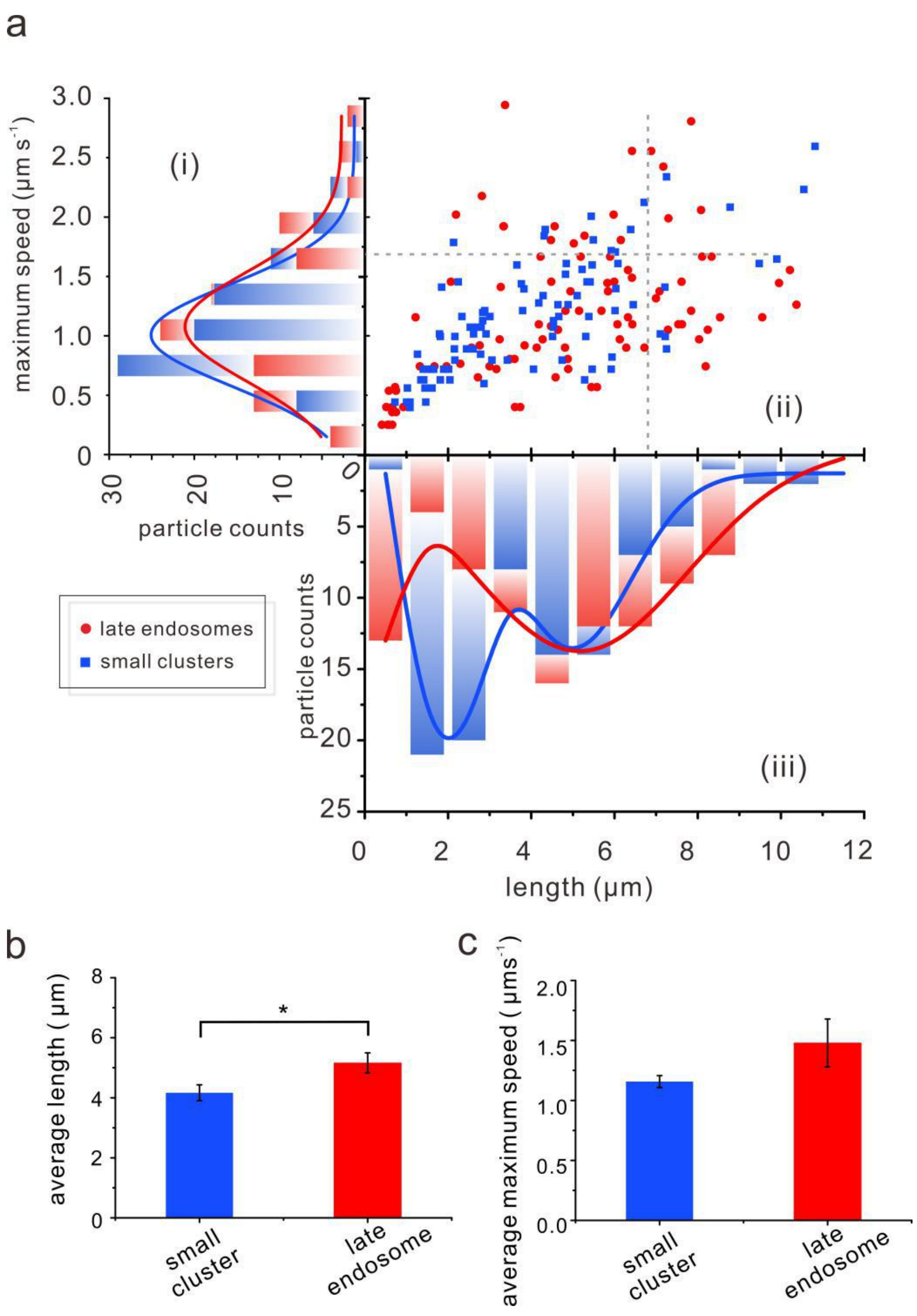

Supplementary Figure 22. Movement of small clusters and late endosomes in cells. (a-ii) Scatter plots showing passage length and maximum of frame to frame instantaneous speed for each small cluster (blue) and late endosome (red); (a-i) histogram showing the distribution of speed; (a-iii) histogram showing the distribution of passage length. (b) Average passage length and (c) average maximum speed of small clusters and late endosomes (data obtain from single-particle analysis shown in

(a), and were presented as the mean ± SEM. *P<0.05 according to two-tailed two-sample *t* test). Data were collected from 100 randomly selected spots in three independent experiments for each group.

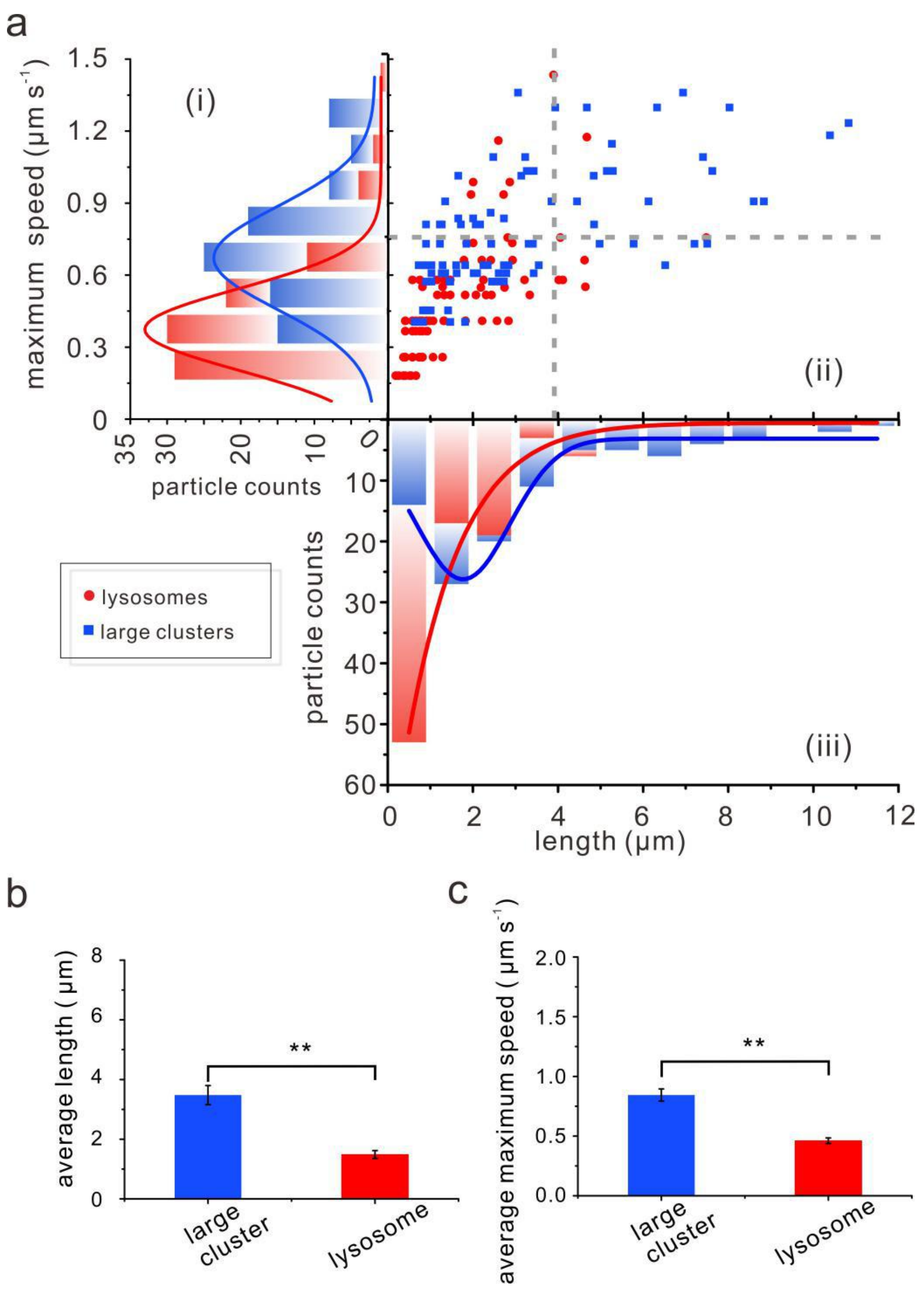

Supplementary Figure 23. Movement of large clusters and lysosomes in cells. (a-ii) Scatter plots showing passage length and Maximum of frame to frame instantaneous speed for each large cluster (blue) and lysosome (red); (a-i) histogram showing the distribution of speed; (a-iii) histogram showing the distribution of passage length. (b) Average passage length and (c) average maximum speed of large clusters and

lysosomes (data obtain from single-particle analysis shown in (a), and were presented as the mean ± SEM. **P<0.01, according to two-tailed two-sample *t* test). Data were collected from 100 randomly selected spots in three independent experiments for each group.

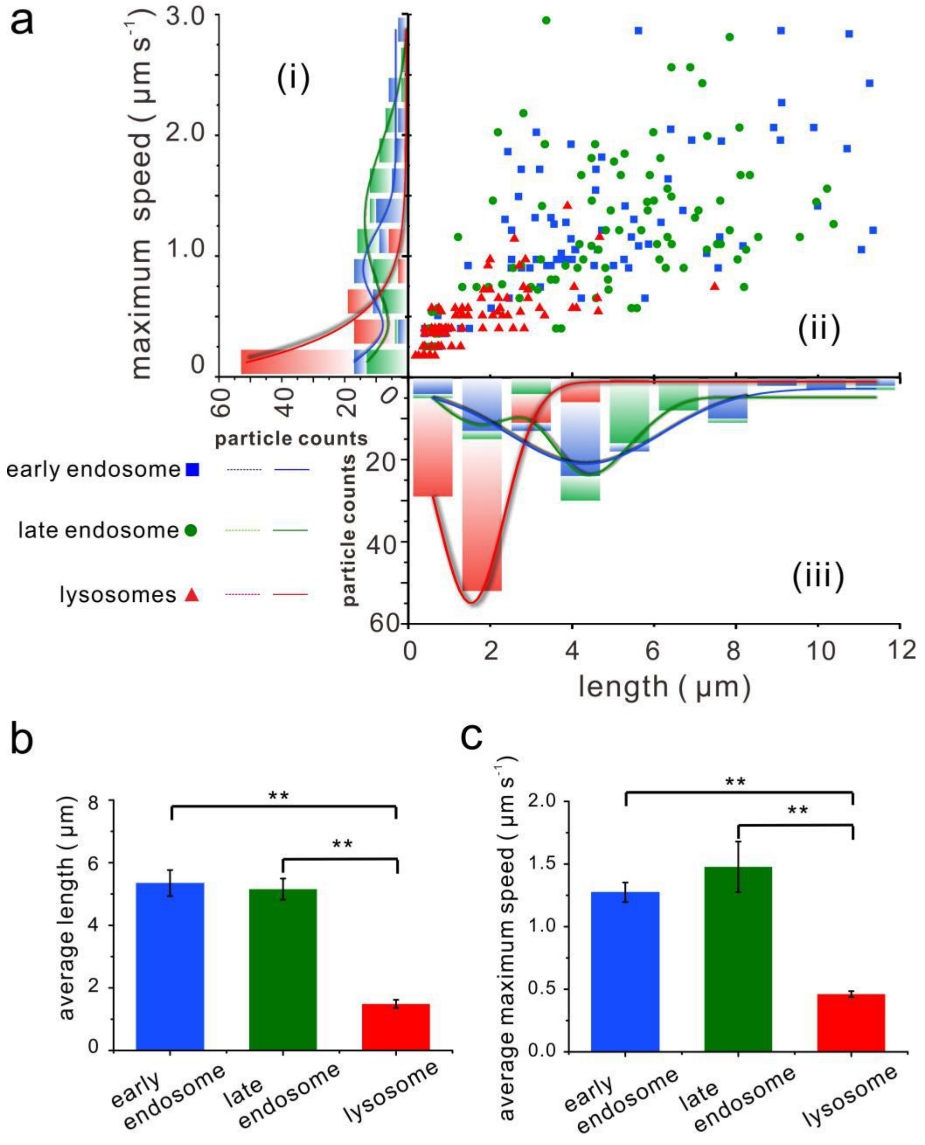

Supplementary Figure 24. Movement of early endosomes, late endosomes and lysosomes in cells. (a-ii) Scatter plots showing passage length and maximum of frame to frame instantaneous speed for each early endosome (blue), late endosome (green) and lysosome (red); (a-i) histogram showing the distribution of speed; (a-iii) histogram showing the distribution of passage length. (b) Average passage length and (c) average maximum speed of early endosomes, late endosomes and lysosomes (data

obtain from single-particle analysis shown in (a), and were presented as the mean ± SEM. **P<0.01, according to two-tailed two-sample *t* test). Data were collected from 100 randomly selected spots in three independent experiments for each group.

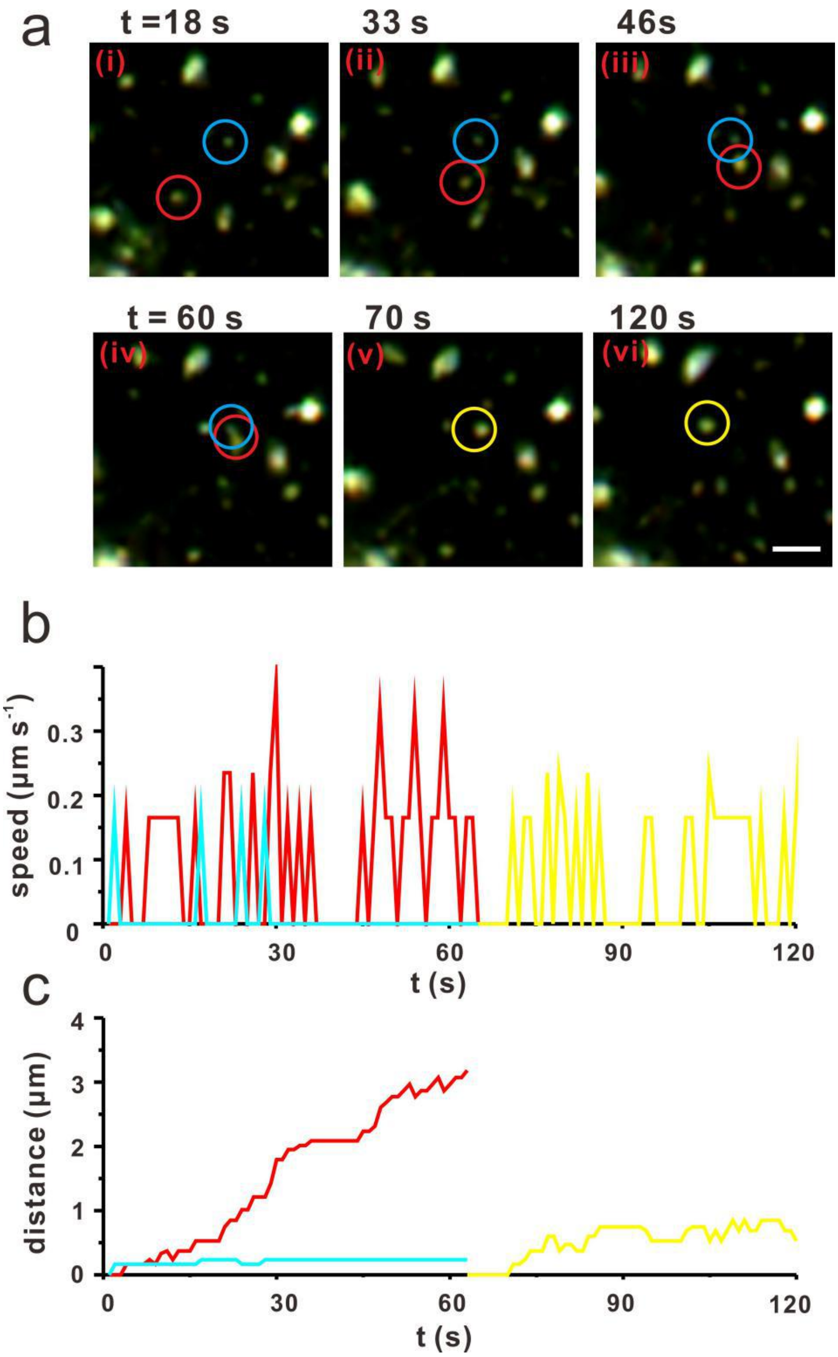

Supplementary Figure 25. Analysis of particle speed and moving distance for the motion I, chase and merge. Scale bar represents 2 μm. See Supplementary Movie 11.

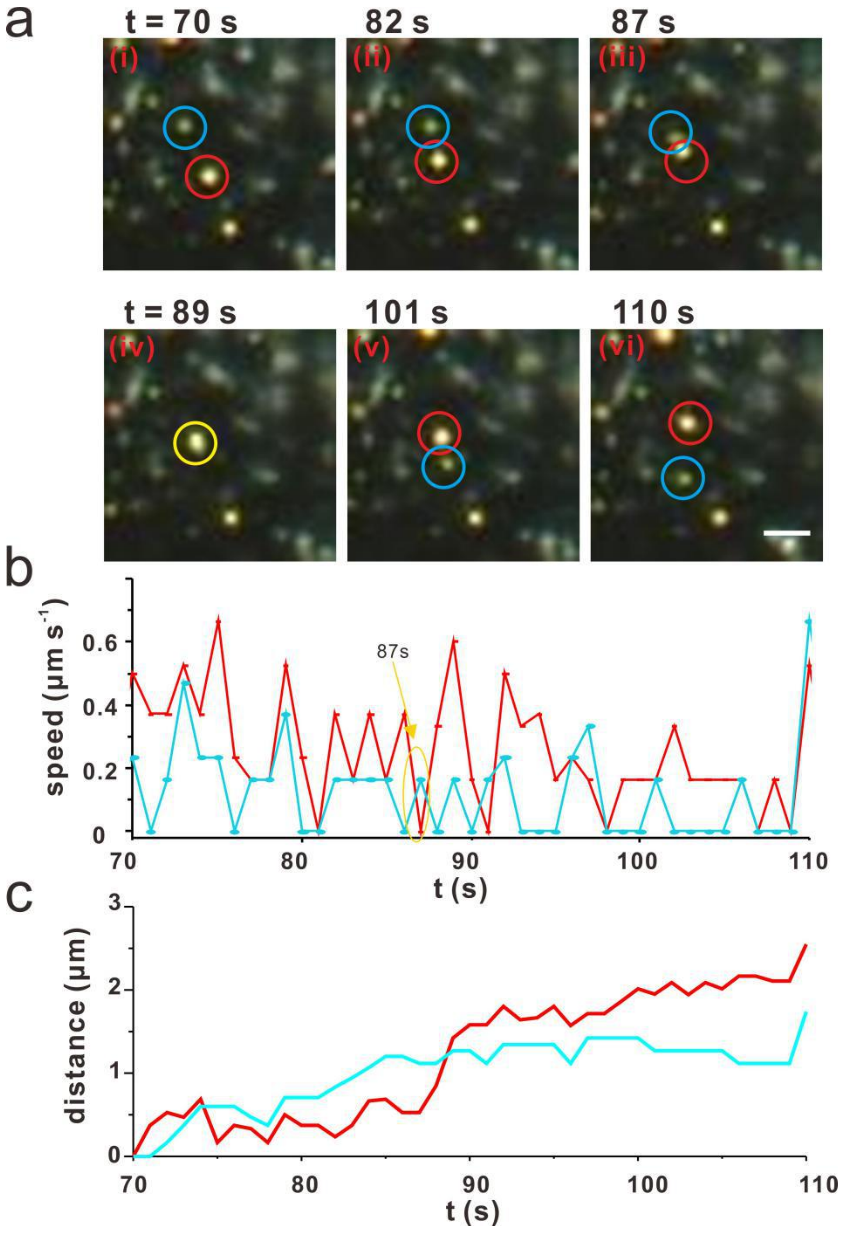

Supplementary Figure 26. Analysis of particle speed and moving distance for the motion II, kiss and run. Scale bar represents 2 μm. See Supplementary Movie 12.

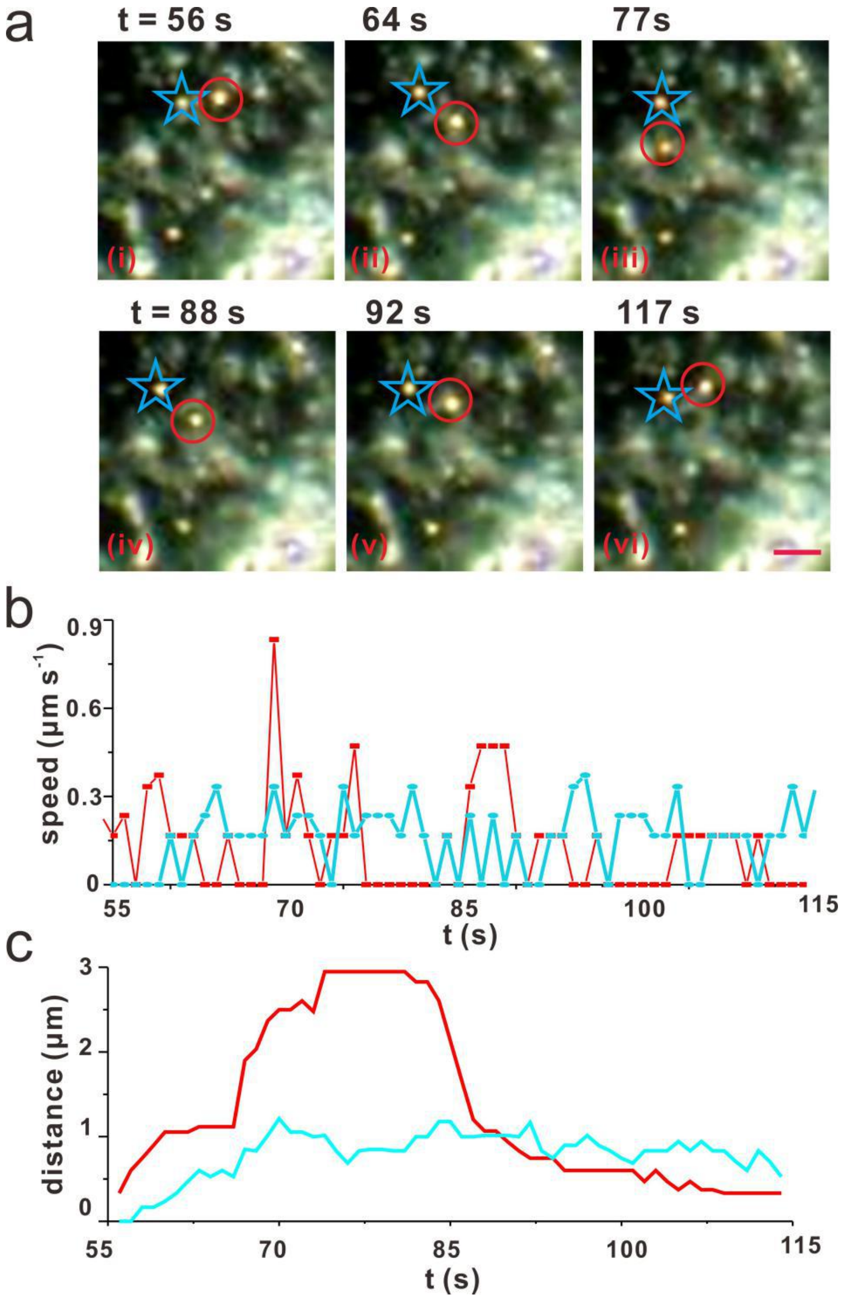

Supplementary Figure 27. Analysis of particle speed and moving distance for the motion III, back and forth. Scale bar represents 2 μm. See Supplementary Movie 13.

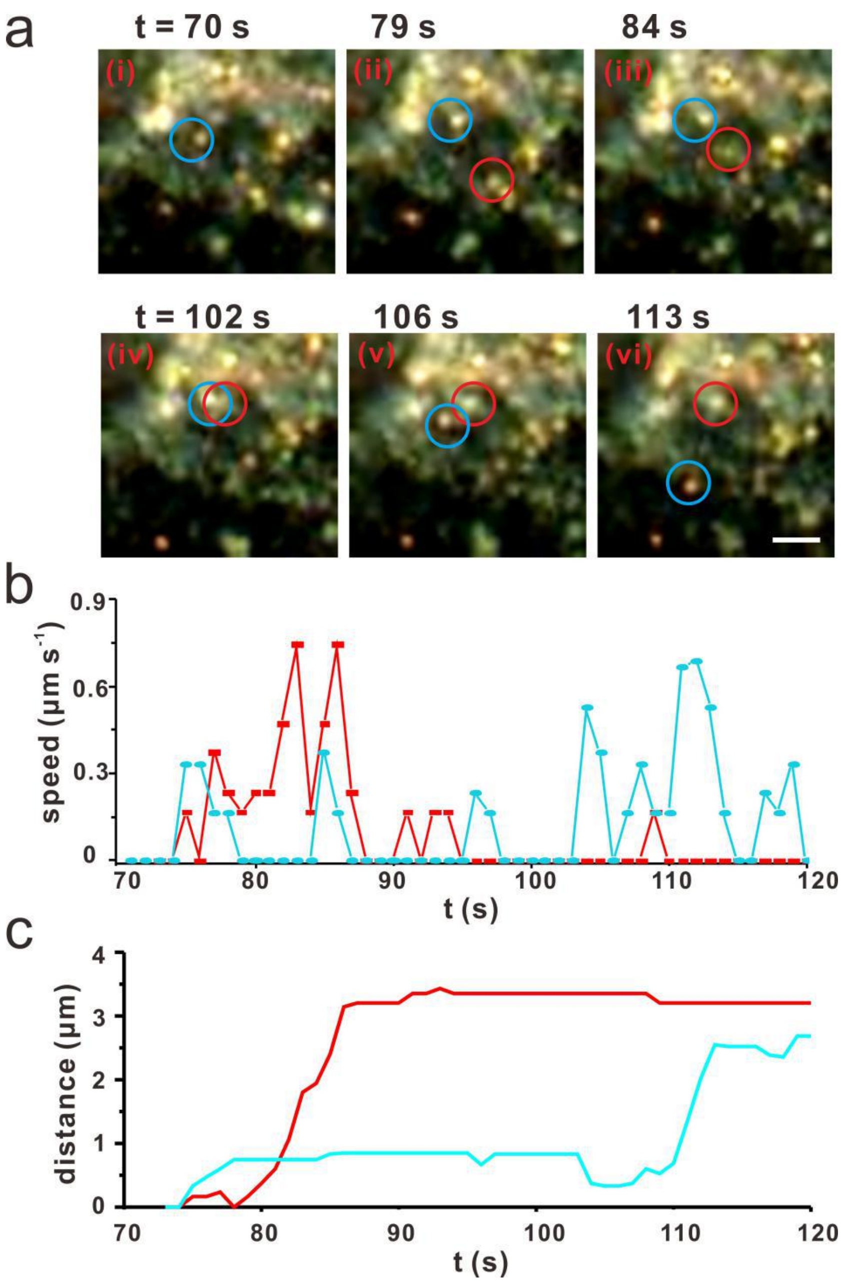

Supplementary Figure 28. Analysis of particle speed and moving distance for the motion IV, stop shot. Scale bar represents 2 μm. See Supplementary Movie 14.

Supplementary Discussion

Endocytic mechanisms

TEM studies provided direct evidence for the endocytic mechanisms. The majority of fPlas-gold was trapped in vesicles, with only few found in the cytosol (Supplementary Figure 13a). Further analysis of these vesicle structures revealed the formation of clathrin-coated pits (CCPs) that connected to the cellular surface via a neck (Supplementary Figure 13b) or an electron-dense clathrin-coated vesicle with an outer diameter around 100 nm (Supplementary Figure 13c); caveosomes with non-electron-dense spherical vesicles with an outer diameter less than 100 nm (Supplementary Figure 13d); and macropinosome ruffles with a diameter larger than 1 μm (Supplementary Figure 13e), suggesting the occurrence of multiple types of internalization pathways including caveole- and clathrin-mediated endocytosis as well as macropinocytosis.

Categorization of fPlas-gold dots with different colors

The green, yellow and bright yellow colors of fPlas-gold in DFM images are the colors that we visualized under the microscope, which are further corroborated by collecting their scattering spectra (see supplementary Figure 7b). We then compared our naked-eye classification of randomly-selected 20 fPlas-gold clusters with the results based on scattering spectra, and found a good agreement (see supplementary Figure 7d).

We also performed finite-difference time-domain (FDTD) simulation and found that the simulated scattering spectra were generally consistent with the measured ones (Supplementary Figure 7c).

Correlative imaging with SEM and DFM established the dependence of the color change on the aggregation states of fPlas-gold. A single particle of fPlas-gold exhibited green color in DFM images, whereas the color of clustered fPlas-gold gradually turned to yellow along with the increased number (n) of particles (from n=1 to n=10) (Supplementary Figure 7a). Yellow spots in DFM images are small clusters containing 2-5 single particles under SEM imaging and bright yellow spots are large clusters containing more than 5 particles.

Next, we performed a quantitative analysis on the correlation between the colors of fPlas-gold in DFM images with their aggregation states. A wide-field DFM image containing approximately 100 particles was recorded (Supplementary Figure 6a). This image was subsequently used as a pattern recognition template during the SEM

analysis to locate and correlate particles of different aggregation states (Supplementary Figure 6b). This study confirmed the robustness of color classification.

Supplementary Methods

Materials

HPLC purified synthetic oligonucleotides were purchased from Takara and used without further purification. Oligonucleotide sequences used in experiments are shown below:

S1: 5'-SH-AAAAA AAAAA GAGCT GCACG CTGCC GTC -3'

S2-CY3: 5'-CY3- GACGG CAGCG TGCAG CTC-3'

$HAuCl_4$ was purchased from J&K Chemicals. Nocodazole, cytochalasin B, chlorpromazine and methyl-β-cyclodextrin were purchased from Sigma-Aldrich. All other chemicals were purchased from Sinopharm Chemical Reagent. All the chemicals were used without further purification.

Preparation of fPlas-gold

Citrate-stabilized 50nm AuNPs were synthesized according to previous literature [1] and characterized using UV-Vis and TEM.

The synthesized aqueous AuNPs were mixed with thiol modified oligonucleotide S1 with a final AuNPs:S1 molar ratio of 1:3000 in milliQ water and incubated at room temperature overnight to form ssDNA-AuNPs. Then PB concentration was adjusted to 10 mM and NaCl concentration was increased to 50 mM. The resultant solution was sonicated for ~10 s followed by a 30 min incubation at room temperature, and this salting process was repeated till NaCl concentration reached 300 mM. The resultant solution was incubated overnight at room temperature and then centrifuged. The supernatant was removed and the precipitates were resuspended in 10 mM PB (pH 7.4). This washing process was repeated for three times and then the ssDNA-AuNPs were resuspended in 1×PBS (pH 7.4) for further uses.

To a 1nM ssDNA-AuNPs solution in 100 mM PBS (pH 7.4), the complementary oligonucleotide sequence S2-CY3 in 100 mM PBS was added with a final AuNPs:S2-CY3 molar ratio of 1:3000. This mixture was incubated at 37 ^{o}C for more than 30 min to yield Cy3 tagged dsDNA-AuNPs (*i.e.* fPlas-gold) then centrifuged and washed following procedures described above. The final product was resuspended in

1×PBS (pH 7.4) for further uses.

Internalization of fPlas-gold

HeLa cells were cultured in 60 mm Petri dishes overnight, thenthe culturing supernatant was removed and the cells was washed with 1×PBS buffer (pH 7.4) twice. The cells were subsequently incubated for different time in fresh DMEM medium with 0.1 nM fPlas-gold. The uptake process was stopped by washing cells with PBS buffer twice, and the cells were cultured in fresh DMEM again for DFM imaging. To investigate effect of temperature and pharmacological inhibitors, cells were incubated in four different conditions for 30 min (a, 4 °C; b, 10 μg ml^{-1} chlorpromazine; c, 2.5 mM MβCD; d, fresh DMEM medium), respectively, then exposed to 0.1 nM fPlas-gold for 1 hour before DFM imaging.

For ICP-AES measurements, cells with internalized fPlas-gold were washed with 1×PBS three times and then trypsizined and centrifuged at 5000 rpm for 3 min. Cell pellets were digested with aqua regia ($HCl:HNO_3$=3:1) at room temperature overnight and the content of Au-197 of resultant solution was measured with an Optima 8000 ICP-OES spectrometer (PerkinElmer).

For TEM imaging, cells with internalized fPlas-gold were washed, trypsizined and centrifuged. Then cells were resuspended and fixed with 2.5% glutaraldehyde in 1 ×PBS buffer (pH 7.4) and stained with 1% OsO_4 at 4 °C. After gradual dehydration with ethanol and acetone, cell pellets were embedded in Epon 812 resins (Electron Microscopy Science) and sliced to pieces with a thickness of 70nm then stained with uranyl acetate. Images of cell slices were taken with a FEI Tecnei G2-205 Twin transmission electron microscope using a beam voltage of 80 kV.

Co-localization of fPlas-gold with endosomes and lysosomes

For endosome staining, HeLa cells were cultured in 35 mm Petri dishes overnight. Lipo solution was prepared by diluting lipofectamin®3000 in opti-MEM Medium; master solution was prepared by diluting DNA (Rab5-GFP for Early Endosomes and Rab7 for Late Endosomes) in opti-MEM Medium and add P3000® Reagent to master solution. Then, we mixed lipo solution and master solution well, incubated them for 5 minute, and then added complex solution in fresh MEM Medium.The culturing supernatant of Hela cells was removed and the cells was washed with 1×PBS buffer (pH 7.4) twice. Then the cells were incubated in mixed MEM Medium for above 24 hours. HeLa cells were cultured in 35 mm Petri dishes overnight. Then the culturing supernatant was removed and the cells was washed with 1×PBS buffer (pH 7.4) twice. The cells were subsequently incubated for different time in fresh MEM medium with 0.1 nM fPlas-gold. Finally, to observe co-localization of fPlas-gold with endosomes

by using confocal and DFM, cells were washed with 1×PBS buffer and fixed in 4% (wt/vol) paraformaldehyde and 4% (wt/vol) sucrose in 1×PBS buffer at room temperature for 20 min. To observe dynamic co-localization of fPlas-gold with endosomes, cells were washed with 1 ×PBS buffer (pH 7.4) and cultured in fresh MEM for confocal imaging.

For lysosome staining, cells were first incubated with 0.1 nM fPlas-gold for different time, then washed and incubated in fresh medium containing 50 μM probes (LysoTracker® Green DND-26, Invitrogen) for 5 min. Finally cells were washed with 1 ×PBS buffer (pH 7.4) and cultured in freshMEM for confocal imaging.

Co-localization of fPlas-gold with tubulin

HeLa cells were incubated with 0.1 nM fPlas-gold for 6 h, washed with 1×PBS buffer and fixed in 4% (wt/vol) paraformaldehyde and 4% (wt/vol) sucrose in 1×PBS buffer at room temperature for 20 min. After blocking with PBS containing 6% (wt/vol) BSA and 0.25% (vol/vol) Triton X-100 for 45 min,cells were stained with anti-α-tubulin Mouse IgG (B-7) (sc-5286, Santa Cruz) followed by Goat anti-Mouse Chromeo 488 IgG (H&L) (ab60313, Abcam), then washed for confocal imaging. Cover slips were air dried before experiments.

Single-particle tracking of fPlas-gold using fluorescence imaging

Cells were incubated in fresh MEM medium containing 0.1 nM fPlas-gold for 30 min, then washed and visualized using TIRF microscope. To investigate effect of pharmacological inhibitors on particle movement, HeLa cells were incubate with 0.1 nM fPlas-gold for 6 h, and then incubated with media containing 60 μM nocodazole and 20 μM cytochalasin B for 30 min for confocal imaging, respectively. The drugs were maintained in the cell culture throughout the experiments.

To observe the movement of fPlas-gold along microtubules, HeLa cells were first incubated with staining solution of tubulin (CellLight® Tubulin-GFP, BacMam 2.0, Life Technologies, 15 μL in 200 μL medium) overnight, then incubated with 0.1 nM fPlas-gold for 2 h and washed before confocal imaging.

Single-particle tracking of fPlas-gold using DFM imaging

Cells were incubated in fresh MEM medium containing 0.1 nM fPlas-gold for 1 h, washed and then visualized using DFM.

Image analysis

Fluorescence images were first analyzed using ImageJ software (US National

Institutes of Health). To quantify the co-localization efficiency of two fluorescent signals, *tMr* values(the thresholded Mander's coefficients) indicating the percentage of Cy3 signals co-localized with green signals in merged images were calculated. Values represent mean ± SE based on analysis of randomly selected 10 cells from three independent experiments. For single particle tracking, the trajectories of Cy3 signals were built by pairing spots in each frame using single-particles tracking plug-in of ImageJ.

Speed calculation and mean square displacement (MSD) analysis were performed using user-written program with MATLAB (The MathWorks) software. MSD data of each particle was calculated following formula shown below [2]:

$$MSD(n\Delta t) = [\frac{1}{N-n}\sum_{i=1}^{N-n}(\vec{r_i} - \vec{r_{i+n}})^2],$$

in which Δt is the time interval between two successive recorded images, N is the total number of frames, n is a positive integer that determines the time increase, and r is displacement. The upward and downward relationships of the MSD over time plots indicate the movement is the manner of directed motion and anomalous diffusion, respectively.

Supplementary References

[1] Bastus, N.G., Comenge, J. & Puntes, V. Kinetically controlled seeded growth synthesis of citrate-stabilized gold nanoparticles of up to 200 nm: size focusing versus Ostwald ripening. *Langmuir* **27**, 11098-11105 (2011).

[2] Schütz, G. J., Schindler, H. & Schmidt, T. Single-molecule microscopy on model membranes reveals anomalous diffusion. *Biophysical Journal* **73**, 1073-1080(1997).
